# Molecular Analysis of Thymopentin Binding to HLA-DR Molecules

**DOI:** 10.1371/journal.pone.0001348

**Published:** 2007-12-26

**Authors:** Zuojia Liu, Xiliang Zheng, Jin Wang, Erkang Wang

**Affiliations:** 1 State Key Laboratory of Electroanalytical Chemistry, Changchun Institute of Applied Chemistry, Chinese Academy of Sciences, Changchun, Jilin, China; 2 Department of Chemistry and Physics, State University of New York, Stony Brook, New York, United States of America; Vanderbilt University, United States of America

## Abstract

Thymopentin (TP5) triggers an immune response by contacting with T cells; however the molecular basis of how TP5 achieves this process remains incompletely understood. According to the main idea of immunomodulation, we suppose that it would be necessary for TP5 to form complex with human class II major histocompatibility complex DR molecules (HLA-DR) before TP5 interacts with T cells. The uptake of TP5 by EBV-transformed B cells expressing HLA-DR molecules and the histogram of fluorescence intensities were observed by using fluorescent- labeled TP5, testifying the direct binding of TP5 to HLA-DR. The binding specificity was confirmed by the inhibition with unlabeled TP5, suggesting the recognition of TP5 by HLA-DR. To confirm the interaction between TP5 and HLA-DR, the complex formation was predicted by using various modeling strategies including six groups of trials with different parameters, alanine substitutions of TP5, and the mutants of HLA-DR. The results demonstrated that TP5 and its alanine substitutions assumed distinct conformations when they bound to HLA-DR. The observation further showed that there was flexibility in how the peptide bound within the binding cleft. Also, the molecular analysis supplemented a newly important discovery to the effect of Val anchor on TP5 binding HLA-DR, and revealed the important effects of Glu11 and Asn62 on the recognition of TP5. These results demonstrated the capability of TP5 to associate with HLA-DR in living antigen presenting cells (APC), thereby providing a new and promising strategy to understand the immunomodulation mechanism induced by TP5 and to design potential immunoregulatory polypeptides.

## Introduction

Thymopentin (TP5) is a synthetic pentapeptide, corresponding to position 32∼36 of thymopoietin [1]. TP5 exhibits a similarly biological activity as thymopoietin responsible for phenotypic differentiation of T cells and the regulation of immune systems [2]. It had been recognized as an immunomodulator for the treatment of primary immunodeficiencies, such as AIDS [3], rheumatoid arthris (RA) [4] and autoimmune diseases [5] etc. Although the biological role of TP5 has been well elucidated by making contact with T cells, relatively few efforts have been made to clarify the refined mechanism of its action. For the standard paradigm of T-cell mediated immune response, T cell receptors (TCRs) only recognize foreign antigens stably bound to MHC molecules [6]–[10]. Recently, it had been shown that human CD 4 T cells expressed functional class II major histocompatibility complex molecules (MHC II) [11]. Thus, we deduce that it would be necessary for TP5 to form complex with MHC II molecules before it interacts with T cells.

MHC II molecules are proteins anchored in the cell membrane of APC, where they present antigenic peptides to CD4 positive T helper cells [12], [13]. Recent advances had provided insights into how MHC interacted with peptides [14]–[22] and a rationale to predict optimal epitopes of MHC-binding [23], [24]. It is important to note that most of the well-known ligands were derived from naturally MHC-bound peptides and T-restricted epitopes. For synthetic peptides known as clinical drugs, there are few reports on their direct binding MHC in living APC.

In the present study, we have established combined experimental and computational strategies to verify the hypothesis of the complex formation of MHC II/TP5. Taking advantage of confocal-laser scanning microscopy (CLSM) and flow cytometry (FCM) techniques, we examined the binding of fluorescent-labeled TP5 to HLA-DR in living APC *in situ* with an apparent dissociation constant (*K*_d_) of 7.2×10^−6^ M. Furthermore, the binding specificity was tested by competitive binding assay with unlabeled TP5. The molecular modeling of the interaction between ligands and receptors demonstrated that TP5 and its alanine substitutions adopted distinct conformations when they bound to HLA-DR. The observation further showed that there was flexibility in peptide binding with MHC II binding cleft. More importantly, the molecular analysis supplemented a newly important discovery to the effect of Val anchor on TP5 binding HLA-DR. Also, the molecular analysis revealed the key effects of Glu11 and Asn62 on the recognition of TP5 based on the mutants of HLA-DR. The study provides a better understanding to the mechanism of interaction between TP5 and TCRs and a rational strategy to design TP5 analogs.

## Results

### Uptake of FITC-labeled TP5 by EBV-transformed B cells

**Figure 1:**
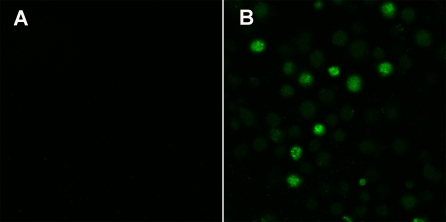
Uptake of FITC-labeled TP5 by EBV-transformed B cells. Confocal images were taken in living cells in the absence (A) and presence (B) of FITC-labeled TP5. The scale bar corresponds to 20 µm.

To validate the ability of FITC-labeled TP5 to load on EBV-transformed B cells expressing HLA-DR, a qualitative CLSM assay was used to examine the fluorescent signal of EBV-transformed B cells. The surface fluorescence was hardly observed from the cells in the absence of FITC-labeled TP5 at the excitation of 488 nm (Fig. 1A). In sharp contrast to this observation, the strong surface fluorescence was found for the cells in the presence of FITC-labeled TP5 at the same conditions (Fig. 1B). These findings indicated that the green fluorescence could attribute to the loading of FITC-labeled TP5 onto the EBV-transformed B cells.

### Direct binding of FITC-labeled TP5 to EBV-transformed B cells

**Figure 2:**
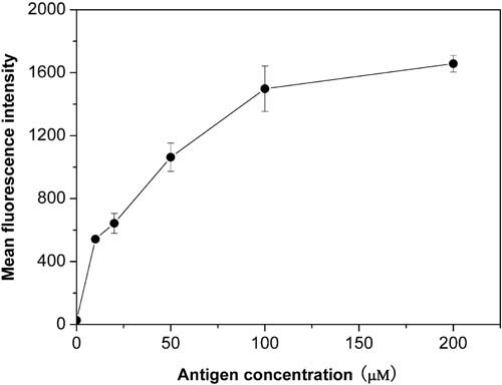
Direct binding of FITC-labeled TP5 to EBV-transformed B cells. The mean binding data for various concentrations of FITC-labeled TP5 were shown as mean fluorescence intensities of triplicates±SD.

**Figure 3:**
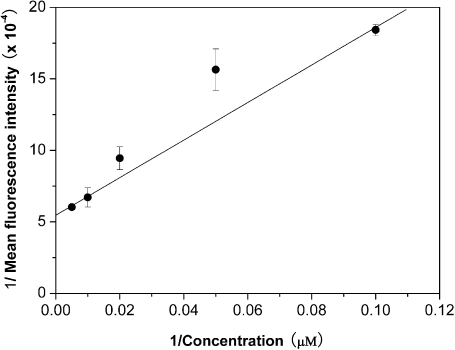
Double-reciprocal plot of the equilibrium binding data. Representative binding data for various concentrations of antigen were expressed as mean fluorescence intensities of triplicates±SD.

**Figure 4:**
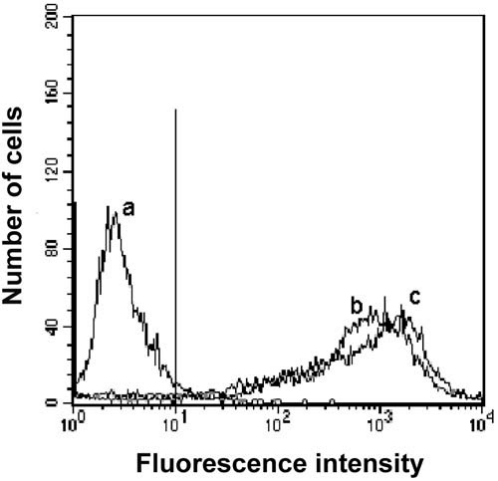
The binding of FITC-labeled TP5 onto the surface of EBV-transformed B cells. EBV-transformed B cells were incubated with PBS alone (trace a), FITC-labeled TP5 (trace b) and mAb-DR FITC (trace c), and then subjected to flow cytometry. The vertical line was the boundary between bound and unbound cells.

Although the uptake of FITC-labeled TP5 in EBV-transformed B cells was confirmed, the correlation between the binding affinity of FITC-labeled TP5 to EBV-transformed B cells and the fluorescence intensity in cells was not showed clearly. Therefore, a quantitative flow cytometric assay was used to get a better insight into the binding affinity of TP5 to HLA-DR on the surface of EBV-transformed B cells. As shown in Fig. 2, the binding of FITC-labeled TP5 to EBV-transformed B cells was in a dose-dependent manner, and the equilibrium could be achieved at 100 µM of antigen. An apparent *K*_d_ of 7.2×10^−6^ M was obtained from the double- reciprocal plot of the equilibrium binding data (Fig. 3), implying that TP5 markedly bound to HLA-DR. Simultaneously, a correlation with *R^2^* = 0.9903 and a slope of 0.01311 were obtained. We then compared the specific binding of FITC-labeled TP5 to HLA-DR. In Fig. 4, the histogram of fluorescence intensities indicated that almost the same extent cells (94% vs. 95%, respectively) were stained by FITC-labeled TP5 and mAb-DR FITC above the background, with an approximately 3 orders of magnitude increase in mean fluorescence intensity (MFI). The fluorescent signal showed the strongly direct binding of FITC-labeled TP5 to HLA-DR expressed on the surface of EBV-transformed B cells.

### Competitive binding assay

**Figure 5:**
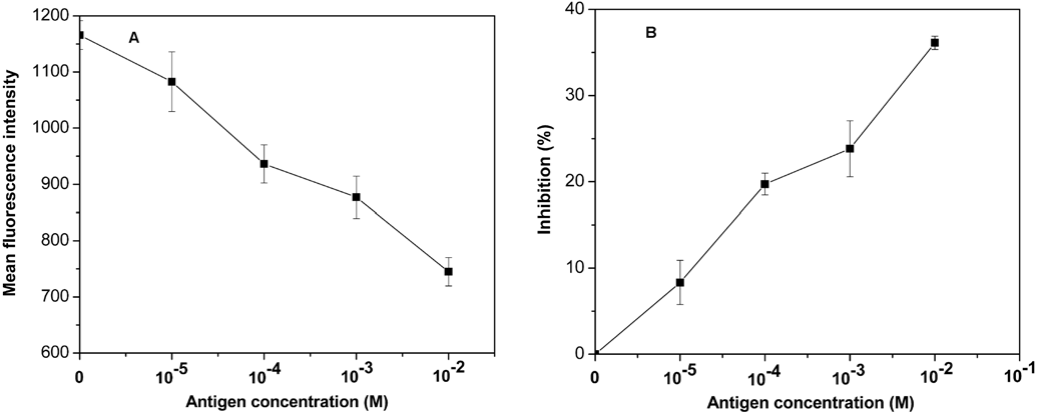
Inhibition of the binding of mAb-DR FITC by unlabeled TP5. Results were shown by the decrease of MFI (A) and the percent inhibition of MFI (B). The data points shown were the mean values of triplicates±SD.

Next, we examined whether the blocking of HLA-DR on the surface of APC would lead to the inhibition of mAb-DR binding. A distinct change of fluorescence intensity was observed when unlabeled TP5 was preincubated with EBV-transformed B cells, followed by incubation with mAb-DR FITC. With increasing the concentrations of TP5, the fluorescence intensity was shifted to low level of MFI (Fig. 5A). The change of MFI was dependent on the concentrations of TP5 used in the competitive binding assay (Fig. 5B). The reduction of MFI indicated that TP5 bound to HLA-DR and inhibited the binding of mAb-DR to HLA-DR. The competitive binding assay implied that the fluorescence intensity was correlated with the amount of HLA-DR on the surface of APC. The observation further indicated that TP5 restrained the binding of mAb-DR to HLA-DR molecules.

### Molecular modeling of the interaction between TP5 and HLA-DR

The first five dockings were terminated based on the endpoint of the number of energy evaluations listed in Table 1. As a result, the maximum number of generations was less than 27000 in these runs. Whereas, the sixth docking was terminated based on the endpoint of the maximum number of generations (27000), and the number of energy evaluations was 100 million. From the current data, it could be clearly seen that the number of energy evaluations had an influence on the docking energy generated from six trials. We found that the last trial converged to a stably native binding mode. We also found the binding modes with lowest energy generated from other 5 trials shared some common features with the native binding mode in the last trial. This illustrated that the common features shared by different trials might be crucial for the binding of TP5 to HLA-DR. This means that few stable binding conformations could be found below 100 million energy evaluation.

**Table 1:** Docking energies of six groups of trials under different energy evaluations.

Trials	Energy evaluations (×10^6^)	aE_docked_/bE_free _(kcal/mol)
1	5	−15.54/−6.68
2	7.5	−16.95/−7.95
3	10	−16.48/−9.16
4	20	−15.18/−6.02
5	50	−17.21/−9.22
6	100	−17.86/−9.25

a E_docked_ represents the sum of the intermolecular and internal energies of ligands;

b E_free_ represents the free energy evaluated by the method provided in the autodock package.

More importantly, we found that Gln9 and Lys250, locating in the brim of the binding site, were ineffective to direct peptides to bind into the binding cleft. On the contrary, the interactions between peptide and Glu11 and Asn62 played a key role in orientation, suggesting that both the residues were obligatory during the binding of TP5 into the binding cleft.

**Figure 6:**
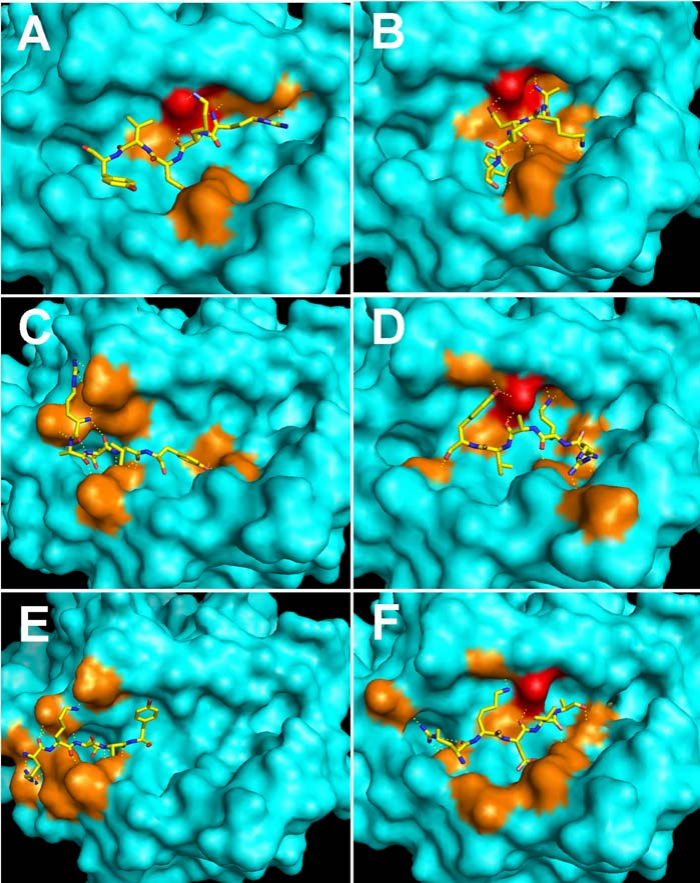
The predicted binding of TP5 and its alanine substitutions to HLA-DR. The key residues (E11, N62) are in red; the other contacted residues are in orange. Peptide variants correspond to A: RKDVY; B: AKDVY; C: RADVY; D: RKAVY; E: RKDAY; F: RKDVA.

On the basis of Autodock3.0.5 method, a certain number of energy evaluations may converge to a correct energy minimum. That implies more members of the populations will be converged to the lowest energy conformation [25]. Thus, if the energy evaluation is further increased, the system will be remained at the same conformation. According to the rule, the maximum number of energy evaluations was set to 100 million for the following calculations. Confirming the complex formation between TP5 and HLA-DR, we assessed the ability of HLA-DR to functionally recognize alanine substitutions of TP5 under the same parameters. The best complexes with the lowest binding energy and the same direction between peptides and the groove were used as the inputs for further minimization. In Table 2, the binding energy of TP5 binding to HLA-DR was the least value among six binding energies. This result means that the most stable complex of HLA-DR/TP5 was formed (Fig. 6A). Remarkably, the substitution of Arg at position 1 considerably reduced the binding affinity. However, as shown in Fig. 6B, the alanine substitution of Arg stretched into the cleft deeply and bound to the same binding site with Glu11 and Asn62, which accounted for its obligatory nature for Arg at position 1 for actual interacting with the receptor. In contrast to this observation, the alanine substitution of Lys at position 2 reduced the binding affinity to the minimum extent; the substitution failed to stretch into the same binding site containing Glu11 and Asn62 instead of projecting out the binding groove (Fig. 6C). In particular, the interactions of Arg and Lys with the binding sites ensured the occurrence of binding and the direction of the N-terminal of the peptide. Whereas, the alanine substitution of Val at position 4 reduced the binding affinity to the maximum extent and bound HLA-DR in a distinct manner from TP5, projecting out the cleft (Fig. 6E). The substitution of Asp and Tyr at positions 3 and 5 resulted in a moderate decrease of binding affinity and variant binding sites without Glu11. Interestingly, both the substitutions bound into the binding cleft (Fig. 6D, F). Examination of the docking results revealed that TP5 was predicted to be optimal for binding into the cleft within the MHC molecules.

**Table 2:** The effect of alanine substitutions of TP5 on binding HLA-DR.

Substitutions	Binding energy (kcal/mol)	Key residues
RKDVY	−8.99	E11, N62
**A**KDVY	−6.31	E11, N62
R**A**DVY	−7.27	
RK**A**VY	−7.18	N62
RKD**A**Y	−6.11	
RKDV**A**	−7.09	N62

As mentioned above, the contacted residues Glu11 and Asn62 played a key role in mediating the binding affinity and orientation (Table 2). In order to verify their effects on the binding between TP5 and HLA-DR, the HLA-DR double mutant, replacing E11 and N62 with alanine (DR11/62), and two single mutants, DRE11A (DR11), and DRN62A (DR62), were used as controls. From Table 3, when TP5 bound to the mutant DR11, the binding energy had a slight change; however, Ala11 completely lost its role as a binding site. Whereas, when TP5 bound to the mutant DR62, the binding energy had a distinct change; Ala62 remained to exert a positive role as a binding site, suggesting that Asn anchor mainly contributed to the binding energy. When the mutant DR11/62 was used, the binding energy increased markedly which was basically equal to the sum of both the values above predicted, demonstrating that Glu11 and Asn62 played their respective role in the double mutant. These data suggested that the complex of HLA-DR/TP5 was the most stable, and further indicated that the molecular modeling was feasible for supporting the assumption of TP5 binding to HLA-DR.

**Table 3:** The effect of the mutants of HLA-DR on the recognition of TP5.

Mutants	Binding energy (kcal/mol)	Key residues
DR	−8.99	E11, N62
DR11	−8.42	N62
DR62	−7.78	E11, A62
DR11/62	−7.46	A62

## Discussion

The role of TP5 in clinical treatment in immune system was well established, however, the refined mechanism of its action is not known in details. According to the main idea of immunomodulation, we suppose that it were obligatory for TP5 to form complex with MHC II before TP5 interacts with TCRs. For class II MHC molecules, they bound peptides with various length [3]. More importantly, a tentative DR motif that governs the fine-specificity of antigen presentation had been well known recently [26]. For TP5, it contains Val and Tyr residues which belong to basic anchor motifs, providing multiple interaction sites. Especially, TP5 possesses a linear peptide whose structural flexibility may allow it to associate with different receptors through conformational changes. According to the rule of set of allowed anchor residues (SAAR) [26], TP5 is theoretically expected to bind HLA-DR.

We report the first direct observation of MHC II/TP5 complex formed in living APC *in situ*. On the basis of the double-reciprocal plot of the dose-dependent fluorescence intensities (Fig. 3), the *K*_d_ (7.2×10^−6 ^M) is close to the reported *K*_d _value [27]. Furthermore, with increasing the concentrations of TP5 in the range of 10^−5 ^M to 10^−2 ^M, the fluorescence intensity decreased (Fig. 5A), reaching 36% inhibition at the concentration of 10^−2 ^M as shown in Fig. 5B. The inhibition was similar to the observation of human EBV-transformed B cells inhibited by anti-HLA-DR antibody reaching 44–52% [28]. The comparability in the level of inhibition could be due to the higher levels of MHC II on the surface of EBV-transformed B cells [29] and the occupied binding sites of MHC II [2]. The main findings indicated that MHC II/TP5 complex was formed by the binding of TP5 to HLA-DR in living cells. The affinity measured using living EBV-transformed B cells are more applicable for functional recognition of MHC II/peptide by T cells.

As far as MHC-binding motifs are concerned, anchors can be divided into two different types: type 1 anchors that contribute to the binding energy of the peptide, and type 2 anchors that contribute to the peptide conformation [30]. Here, we performed molecular modeling evaluations to analyze the interaction between the anchors in the frame of TP5 and the binding sites within the groove of HLA-DR. The alanine substitutions of TP5 were tested for HLA-DR binding affinity. From the binding energies presented in Table 2, it can be observed that the substitution at positions 1 and 4 had a significant effect on the binding affinity; the substitution at other positions resulted in only partial loss of binding affinity. It implied that the substitution of TP5 at positions 1 and 4 formed instable complexes with HLA-DR. Further, the anchor at positions 1 and 4 seemed to be obligatory as type 1 anchors due to decreasing the binding affinity by Ala substitution. It was also noteworthy that the substitution at positions 2 and 4 neither bound to the same binding sites containing Glu11 and Asn62 nor stretched into the binding groove (Fig. 6C, E). This observation suggested that anchors at positions 2 and 4 mainly exerted an effect on peptide conformation, and further implied that the conformation of DR-bound peptide should be very similar. With regard to positions 3 and 5, the substitution by Ala resulted in a moderate binding affinity decrease and variant binding sites containing Asn62 without Glu11, suggesting that peptides with several anchors in frame were more likely to bind to a particular binding site than those lacking partial anchors in frame. The variation of binding affinity showed that anchors at positions 1, 3 and 5 in the frame of TP5 actually interacted with the receptor HLA-DR; however, anchor at position 2 primarily involved in steric constraints. As for the Val at position 4, although the amino acid served as type 2 anchors identified in previous study [31], the contribution as type 1 anchors is more in the present study. This observation exhibits an obligatory role for Val anchor which is essential for HLA-DR/peptide interaction. The functional analysis of TP5 is basically consistent with previous study on the role of each amino acid in frame [31] other than supplementing an important discovery to the effect of Val on TP5 binding receptor as type 1 anchors.

The alanine-substituted peptide variants bound less well to HLA-DR than TP5 did, suggesting that Ala in the substitutions exerted its effect indirectly by inducing a conformational change. Especially, substitution of the N- and C-terminal residues with Ala had revealed some unpredictably conformational changes both in central peptide residues and in the MHC other than affecting the stability of the complexes [32], [33]. Thus, the substitution of anchors can alter overall peptide conformation. Simultaneously, as shown in Fig. 6, the binding space of TP5 and its alanine substitutions in binding grooves was larger than that of the longer peptides. Thus, the binding groove likely permits flexibility in peptides binding, which had been demonstrated before [34], [35]. The results provide significant evidences that peptide variants by a single Ala-substituted assume distinct conformations when they bound to HLA-DR. In other words, the results show that there is flexibility in peptide binding within the MHC II binding cleft.

**Figure 7:**
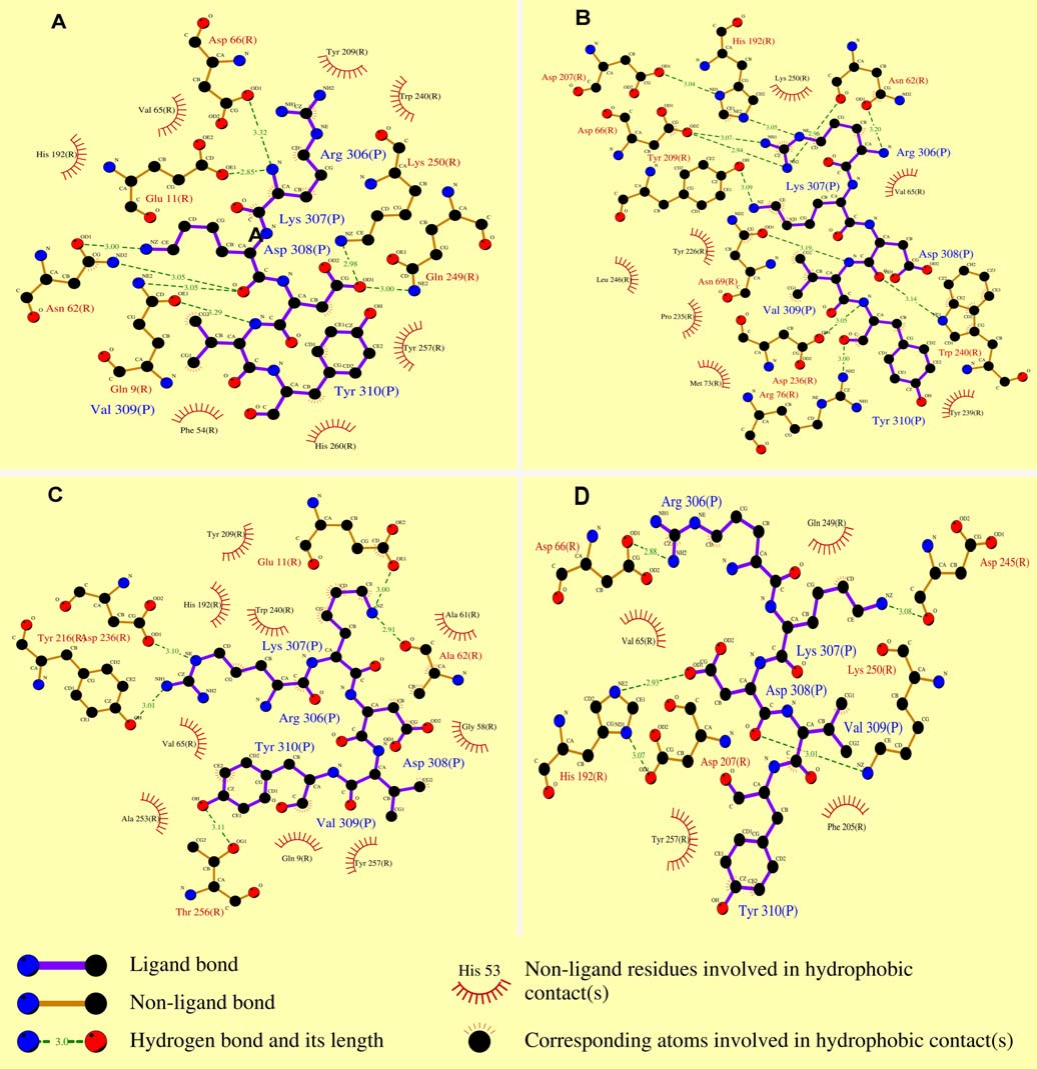
The 2D schemes of the predicted binding of TP5 to HLA-DR and its mutants. The schemes of TP5 binding HLA-DR (A), DR11 (B), DR62 (C), and DR11/62 (D) were resulted from the Ligplot program [42].

In an attempt to gain insight into the interaction pattern, we studied the influence of site-directed mutants of HLA-DR on the peptide binding. The results demonstrated that Glu11 exerted its role of binding site through forming H-bond by the interaction of its side chain with the Lys anchor in the frame of TP5 (Fig. 7A, C). Conversely, Ala11 completely lost the ability to interact with TP5 due to the lack of negative residue in its side chain (Fig. 7B, D), which showed that Glu11 acted as a key effect on the binding between TP5 and HLA-DR. DR62 resulted in significantly lower binding affinity to level comparable to the binding by original DR (Table 3). It can be seen that Asn62 interacted with the Arg and Asp anchors in the frame of TP5 by its side chain (Fig. 7A, B). However, Ala62 completely lost the interaction with TP5 by its side chain oxygen which was replaced by the main-chain oxygen of Ala62 (Fig. 7C, D), suggesting that the interaction by the main chain weakened the binding affinity. The results examining the recognition of the mutations of HLA-DR provided a sufficient basis for the prediction of Glu11 and Asn62 within the groove exerting respective effect on the binding pattern.

The collectively experimental and computational studies supported the hypothesis that TP5 bound to HLA-DR with stable conformation, and clarified the distribution of residue-residue between the peptide and the receptor. This report focused on the exploration of the binding affinity of TP5 to MHC II, which was essential to understand the mechanism of TP5 triggering immune response *in vivo*. More significantly, identification of the interaction between TP5 and HLA-DR will advance our ability to develop polypeptides for the study of MHC class II-restricted CD4+ T lymphocyte responses.

## Materials and Methods

### Peptide synthesis and conjugation with FITC

TP5 was synthesized and purified as described previously [36]. Conjugation of TP5 with Fluorescein isothiocyanate (FITC) (Sigma, St.Louis, USA) was performed and purified as described previously [37]. The identity of FITC-labeled TP5 was conducted by laser-induced fluorescence spectral analysis (PTI, London Ontario, Canada).

### Cell culture

EBV-transformed B cells expressing HLA-DR molecules were maintained in RPMI 1640 containing 2 mM L-glutamine (HyClone Laboratories, UT, USA) supplemented with 10% standard FBS (HyClone Laboratories, UT, USA) at 37°C and in 5% CO_2_. Expression of HLA-DR was verified using mAb-DR L243 labeled with FITC (Pharmingen, San Diego, USA), by incubation for 45 min on ice followed by CLSM and FCM.

### Cellular peptide loading assay

We harvested 3×10^5^ cells, washed twice with phosphate-buffered saline (PBS) buffer (pH 7.4) supplemented with 0.1% BSA and 0.05% NaN_3_. Ten µM FITC- labeled TP5 was added to the treated cells in PBS (pH 7.4) containing 1% BSA (binding buffer), then incubated for 2 h at 37°C. Thereafter, the cells were washed twice and resuspended in 500 µl PBS, followed by CLSM analysis using Leica Microsystems (TCS SP2, Heidelberg, Germany).

### Direct binding of FITC-labeled TP5 to EBV-transformed B cells

EBV-transformed B cells (3×10^5^ per sample) were incubated with various concentrations of FITC-labeled TP5 in binding buffer for 2 h at 37°C. Thereafter, the cells were washed twice and resuspended in 500 µl PBS, followed by flow cytometric analysis using a FACSCan analyzer (Becton Dickinson Immunochemical Systems, Mountain View, USA). A total of ten thousand events were acquired for each sample. The MFI was used to evaluate the binding ability of FITC-labeled TP5 to APC.

### Competitive binding assay

EBV-transformed B cells (3×10^5^ per sample) were preincubated in the presence of varying concentrations of TP5 in binding buffer for 2 h at 37°C. Then, the cells were washed and resuspended in 50 µl binding buffer, followed by incubation with 20 µl mAb-DR L243 labeled with FITC for each sample for 45 min on ice in dark. Thereafter, cells were washed twice and analyzed by FCM. In each analysis, ten thousand cells were examined. Percent inhibition was calculated as [(1–MFI in the presence of TP5/MFI in the absence of TP5)×100%].

### Molecular modeling

Both the numbers of energy evaluations and generations are very important factors for the time that will be consumed and the accurateness of the results that will be got. The docking calculations will be stopped if anyone of the parameters is reached whichever first comes. In this case, to determine the effect of the number of energy evaluations, six groups of trials were performed with 100 runs (Table 1). Six trials with different energy evaluation parameters were chosen for the purpose of exploring which choice could lead to the final binding mode. The maximum number of generations was set to default (27000) so that the docking calculation will be stopped based on the number of energy evaluations. The maximum number of energy evaluations was set to 100 million; the other parameters were defaults. We applied the populations of 300 individuals to 100 independent runs. In addition, the random docking method was applied to predict the complexes between the receptors and the ligands in the Autodock.

Further, the two-step protocol was applied to confirm the complex formation and to examine the interaction between TP5 and HLA-DR. In the present modeling system, HA peptide/HLA-DR complex (PDB code 1J8H) was used as the reference. The structure of TP5 was achieved based on the crystal nonapeptide by mutating method [38]. The ligand TP5 was prepared using AutoDockTools. Here, Amber-based “Kollman” partial atomic charges and solvation parameters were applied to the atoms of the receptor (HLA-DR) using the AutoDockTools software package. Kollman charges were assigned to the atoms of the ligand.

In the first phase, TP5 was docked into the binding site by means of the package Autodock 3.0.5 [39]. A three-dimensional grid was created with 0.375 Å spacing with a 126 Å×126 Å×76 Å grid box by the AutoGrid program for the binding site of the receptor, which was used to evaluate the binding energy between the inhibitor and the protein. Lennard-Jones parameters 12–10 and 12–6 were approximately used to simulate H-bonds and Van der Waals interactions, respectively. The distance- dependent dielectric permittivity of Mehler and Solmajer was used for the calculation of the electrostatic grid maps [40]. The Lamarckian genetic algorithm, the pseudo-Solis, and Wets methods were applied for minimization by using defaults [39]. Solis and Wets local searches were applied with a probability of 6%. A crossover rate of 80% was used to generate new docking solutions for subsequent generations, and one solution from each generation was propagated to the next generation. Random starting positions on the entire protein surface, random orientations, and torsions, were used for the ligand. Evaluation of the results was performed by sorting the binding energy predicted by docking conformations. A cluster of analysis based on the root mean square deviation (rmsd) value, referring to the starting position of the ligand, was performed subsequently.

In the second phase, the lowest-energy complex predicted by molecular docking was subjected to 1000 steps of energy minimization by using the perl script of minAmber MMTSB_tool for removing clashes and refining complex docked by using an Amber-based procedure [38]. Namely, we performed a vacuum minimization of the given structure over 700 steps of final conjugate gradient minimization with 300 steps of initial steepest descent minimization. As a somewhat realistic but very fast method for representing aqueous solvent, we used a distance dependent dielectric function with an epsilon value of 4. The non-bonded cutoff radius was set to 16 Å. With respect to the above minimized complex, the evaluation of the predicted binding energy was made by using the package Dcomplex [41], which is the program for predicting the binding affinities of protein-protein and protein-peptide complexes based on the Distance-scaled Finite Ideal-gas Reference (DFIRE) energy function. The determination of the interactions between ligands and receptors was illustrated by using the package Pymol based on the minimized structures [42].

Next, we studied the dockings for alanine substitutions of TP5 through scanning the alanine on each position for the same receptor (HLA-DR). Moreover, the HLA-DR double mutant DR11/62 and two single mutants DR11, and DR62, were used by molecular modeling method for the same ligand TP5. All preparations and parameters were consistent with the contents above described.
